# Sedentary patterns, physical activity and health-related physical fitness in youth: a cross-sectional study

**DOI:** 10.1186/s12966-017-0481-3

**Published:** 2017-03-04

**Authors:** Pedro B. Júdice, Analiza M. Silva, Juliane Berria, Edio L. Petroski, Ulf Ekelund, Luís B. Sardinha

**Affiliations:** 1 Exercise and Health Laboratory, CIPER, Faculdade de Motricidade Humana, Universidade de Lisboa, Estrada da Costa, Cruz-Quebrada, Lisbon, 1499-002 Portugal; 2 Graduate in Physical Education Program, Kinanthropometry Center and Human Performance, Federal University of Santa Catarina, Santa Catarina, Brazil; 3 Department of Sport Medicine, Norwegian School of Sport Sciences, Oslo, Norway; 4 MRC Epidemiology Unit, University of Cambridge, Cambridge, UK

**Keywords:** Breaks, Bouts, Sedentary behavior, Physical activity, Cardiorespiratory, Adolescents

## Abstract

**Background:**

Strong evidence indicates that moderate-vigorous physical activity (MVPA) is positively associated with fitness in youth, independent of total sedentary-time. Sedentary-time appears negatively associated with fitness only when it replaces MVPA. However, whether different sedentary-patterns affect health-related fitness is unknown.

**Methods:**

The associations between MVPA and sedentary-patterns with physical fitness were examined in 2698 youths (1262 boys) aged 13.4 ± 2.28 years. Sedentary-time (counts · minute^−1^ < 100) and PA were objectively measured by accelerometry. Each break (≥100 counts · min^−1^ < 2295) in sedentary-time and the frequency of daily bouts in non-prolonged (<30 min) and prolonged (≥30 min) sedentary-time were determined. The FITNESSGRAM® test battery was used to assess fitness. A standardized fitness composite-score (z-score) was calculated by summing the individual z-scores of the five tests adjusted to age and sex.

**Results:**

Positive associations between MVPA and fitness were observed in both boys (β = 0.013, 95% CI: 0.005; 0.021) and girls (β = 0.014, 95% CI: 0.006; 0.022), independent of sedentary-patterns. Modest associations were found for the breaks in sedentary-time with fitness (β = 0.026, 95% CI: 0.009; 0.042), independent of total sedentary-time and MVPA in boys. In girls, non-prolonged sedentary bouts were positively associated with fitness (β = 0.014, 95% CI: 0.003; 0.024), independent of total sedentary-time and MVPA.

**Conclusions:**

These results reinforce that, independent of the time and patterns of sedentary behavior, MVPA is consistently associated with fitness in youth. Modest and inconsistent associations were found for sedentary behaviors. Breaking-up sedentary-time in boys and non-prolonged sedentary bouts in girls were positively associated with fitness, independent of total sedentary-time and MVPA. In order to enhance youth’s fitness, public health recommendations should primarily target MVPA, still, suggestion to reduce and break-up sedentary-time may also be considered.

## Background

Physical fitness is an important predictor of health in youth [1, 2], and poor physical fitness seems to be associated with the development of cardio-metabolic risk factors [2, 3], impaired vascular health [4], body composition [5], poorer cognitive control [6], and poor academic attainment [7].

There is evidence suggesting that both moderate and vigorous intensity physical activity (MVPA) is beneficially associated with physical fitness. In the opposite end of the physical activity spectrum, large amounts of time spent sedentary may contribute to increased fatness [8, 9], and impaired fitness in youth [10–13]. A recent meta-analysis [14] found moderate-to-strong evidence for a relationship of overall sedentary time with some fitness indicators [14]. However, these results suggested that sedentary behavior may only be detrimental to fitness when it replaces time spent in MVPA [15–17], and that MVPA is associated with fitness independent of total sedentary time [18]. Additionally, some of the associations observed between sedentary behavior and cardio-metabolic health indicators in youth are markedly less pronounced when taking fitness into account [19, 20], which suggests that sedentariness and fitness may interact.

Youth are spending more than 60% of their waking day sedentary [21], and there is evidence that sedentary behaviors will track during childhood into adulthood [22]. The high prevalence of this deleterious behavior in this population group and acknowledging that Portuguese youth do not reach sufficient PA levels compared with recommendations [23] justify the need for more research in youth. Additionally, recent findings suggest that breaking-up sedentary time and limiting prolonged bouts of sedentary time may be beneficially associated with weight status [24, 25], vascular function [26], and cardio metabolic risk [27]. However, whether specific sedentary patterns, e.g. the frequency of breaks and bouts in sedentary time, i.e. may be associated with fitness levels in youth is less explored [17].

Therefore, the aim of this study was to examine the independent associations between MVPA and sedentary patterns (defined here as breaks and bouts in sedentary time) with health-related fitness in a comprehensive sample of Portuguese youth aged 10–17 years.

## Methods

### Study design and participants

Data were derived from a national wide cross-sectional study aimed to examine PA, physical fitness, overweight/obesity prevalence, and related factors in Portuguese school-age youth. Briefly, data were collected by means of proportional stratified random sampling taking into account the location (region), and the number of students by age and gender in each school, in all mainland Portuguese administrative regions (Alentejo, Algarve, Centro, Lisboa, and Norte). Data from 22,179 youth were collected (89% response rate). All participants aged between 10 and 17 years old with a health status that allowed participation in physical education classes were eligible. Sedentary time and PA were objectively measured in a randomly selected subsample of 3165 youths. Participants that did not comply with accelerometer data collection criteria and missed data on any of the health-related physical fitness components were excluded from the analysis. The final sample consisted of 2698 (85%) youths (1262 boys) age 10–17 years (*M*_age_ = 13.40 ± 2.28).

Data collection were conducted during 2008–2009. Participants were examined during physical education classes by specifically trained physical education teachers. Participants were informed about the objectives of the study and a written informed consent was obtained from their legal guardians. The study was approved by the Ethics Council of the Faculdade de Motricidade Humana, Universidade de Lisboa and was conducted in accordance with the Declaration of Helsinki for Human Studies [28].

### Anthropometry

Participants were weighed to the nearest 0.01 kg while wearing minimal clothes and without shoes, on an electronic scale (Seca, Hamburg, Germany). Height was measured to the nearest 0.1 cm with a stadiometer (Seca, Hamburg, Germany) according to the standardized procedures described elsewhere [29]. Body mass index (BMI) was calculated as body mass (kg)/height^*2*^ (m).

### Sedentary time and PA

Sedentary time and PA were assessed by accelerometry (ActiGraph, GT1M model, Fort Walton Beach, FL). The accelerometer is a small device that measures the acceleration of normal human movements, ignoring high frequency vibrations associated with mechanical equipment. All participants were asked to wear the accelerometer on the right hip, close to the iliac crest, for 4 days. The device activation, download, and processing were performed using the software Actilife (v.6.9.1) (ActiGraph, Fort Walton Beach, FL, USA). The devices were activated on the first day in the morning and data were recorded and posteriorly downloaded into 15-sec epochs. Overall, 15-sec epochs were reintegrated to 60-sec epochs, and activity levels were expressed in terms of counts per minute and intensity thresholds were established according to a previous study [30] using the cut points from Evenson et al. [31]. Apart from accelerometer non-wear time (i.e., when it was removed during sleep and water activities), periods of at least 60 consecutive minutes of zero activity intensity counts were also considered as non-wear time. A valid day was defined as having 600 min (10 h) or more of monitored wear, and all participants with at least three valid days (including one weekend day) were included in the analyses. Information regarding the date (day/month/year) of data collection was recorded and posteriorly treated in order to create a covariate indicating the specific season (spring, summer, autumn, and winter) in which the data resulted from.

Each minute during which the accelerometer counts below 100 was considered sedentary time; total sedentary time was the sum of sedentary minutes while the accelerometer was worn. A break in sedentary time was considered as each interruption in sedentary time in which the accelerometer count raised up to or above 100 counts · min^−1^ and which stayed within the light-intensity PA (LIPA) range (100 to 2295 counts · min^−1^) for at least 1 min.

Data processing also derived the following variables: non-prolonged sedentary bouts (the number of periods with less than 30 continuous minutes in sedentary behavior, with no interruptions allowed) and prolonged sedentary bouts (the number of periods with more than 30 continuous minutes in sedentary behavior, with no interruptions allowed). Accelerometer counts ≥ 100 · min^−1^ were classified as active time, with further differentiation to identify separately LIPA (100 to 2295 counts · min^−1^) and MVPA (≥2296 counts · min^−1^). The difference between LIPA and the daily breaks in sedentary time variable is that whereas LIPA is the total cumulative daily time spent in LIPA per day (minutes · day^−1^), breaks in sedentary time represents the number of times sedentary time were broken by LIPA (breaks · day^−1^).

### Health-related fitness

The FITNESSGRAM® test battery was developed to assess physical fitness among youth with a health-related approach, and it is widely used in some states and school districts in the United States of America [32] and in other countries [18]. Based on the FITNESSGRAM®, youths are stratified as being above or below a predetermined threshold for the healthy fitness zone (HFZ) [32]. The FITNESSGRAM includes assessment of health standards for cardiorespiratory fitness (CRF), body weight, and musculoskeletal function [33].

The specific components of the FITNESSGRAM® are curl-ups (abdominal strength and endurance), push-ups (upper body strength and endurance), sit and reach (flexibility of the hamstrings and the lower back), the Progressive Aerobic Cardiovascular Endurance Run (PACER) test (CRF), and BMI (measure that provides an indication of the appropriateness of a youth’s weight relative to height). A standardized fitness composite score (z-score) was calculated by summing the individual age and sex adjusted z-scores as follows:

$$ \mathrm{Composite}\ \mathrm{z}\hbox{-} \mathrm{score}=\mathrm{z}\hbox{-} \mathrm{Curl}\hbox{-} \mathrm{up}+\mathrm{z}\hbox{-} \mathrm{Push}\hbox{-} \mathrm{U}\mathrm{p}\mathrm{s}+\mathrm{z}\hbox{-} \mathrm{Sit}\ \&\ \mathrm{Reach}+\mathrm{z}\hbox{-} \mathrm{Pacer}+\left(\hbox{-} \mathrm{z}\hbox{-} \mathrm{BMI}\right) $$

When compared to the laboratorial setting, the PACER test has been investigated to be a valid and reliable tool to assess cardiorespiratory fitness [34, 35]. Additionally, the rationale for the tests that are included in the battery to assess strength and flexibility has been widely described [36]. Teacher-obtained health-related large-scale fitness assessment was previously reported to be reliable and valid, and generally independent of potential confounding variables such as student or school characteristics [34].

### Statistical analysis

Statistical analyses were performed using SPSS Statistics for Windows version 21.0, 2012 (SPSS Inc., an IBM Company, Chicago IL, USA). Descriptive analyses included means ± SD for all measured variables. To examine differences in continuous variables between the two sexes, T-Tests were used whereas the qui-square test was used to compare differences in proportions between boys and girls. Interactions between sex and the main covariates for the association with fitness composite score were tested using univariate analysis of variance, and a significant interaction was found for sex with MVPA (*p =* 0.031). Therefore, linear regression models were performed separately for boys and girls to examine associations for total sedentary time, MVPA, daily breaks in sedentary time (breaks · day^−1^), non-prolonged and prolonged sedentary bouts (bouts · day^−1^) with a standardized fitness composite score (z-score), and also with each of the individual FITNESSGRAM® components.

Preliminary models (unadjusted) were developed for the association between all main covariates with the outcomes separately. Analysis with all covariates (MVPA, sedentary time, non-prolonged and prolonged sedentary bouts, breaks in sedentary time, age, and season of data collection) in one model was then performed (adjusted models), and non-significant variables (*P <* 0.1) were eliminated from the final model using backward stepwise approach. Final models were further adjusted for age, season of data collection, and total sedentary time. For example, if only the breaks remained in the model as a significant variable for fitness composite score, the model was adjusted for MVPA, total sedentary time, age, and season of data collection. Conversely, if only MVPA remained in the model, the adjustment would be for total sedentary time, age, and season of data collection. When more than one variable remained in the model, a variance inflation factor (VIF) for each independent variable was calculated to evaluate multicollinearity and a cutoff of <5.0 was considered as an indicator of non-multicollinearity [37]. For all analyses, 5% significance level was adopted, except for the backwards elimination (*p <* 0.1).

## Results

Participants’ characteristics for the whole sample and stratified by sex are shown in Table 1. Boys had significantly higher results in curl-ups, push-ups, pacer 20 m and were more physically active in MVPA (p˂0.001) than girls. Conversely, girls were more sedentary than boys (p˂0.001). On average, boys and girls spent 58% and 61% of their waking hours sedentary, 36% and 35% in LIPA, and 5.5% and 3.6% in MVPA, respectively. Thirty-two % of boys were classified as overweight or obese while 27% of the girls were overweight or obese.

**Table 1:** Participants’ characteristics

	All (*n =* 2698) Mean (SD)	Boys (*n =* 1262) Mean (SD)	Girls (*n =* 1436) Mean (SD)	*p*-value
Age (years)	13.4 (2.3)	13.3 (2.2)	13.5 (2.3)	0.025
Body mass (kg)	50.7 (13.3)	51.4 (14.9)	50.0 (11.8)	< 0.001
Height (m)	1.6 (0.1)	1.6 (0.1)	1.6 (0.1)	< 0.001
BMI (kg/m^2^); HFZ (%)	20.4 (3.8); 70.5%	20.2 (3.8); 67.6%	20.6 (3.8); 73.1%	0.922
Season % (spring; summer; autumn; winter)	35%; 0.7%; 28%; 36%	40%; 0.4%; 29%; 32%	32%; 1.0%; 27%; 41%	< 0.001
Curl ups (repeats); HFZ (%)	36.0 (17.3); 90.4%	40.6 (18.0); 92.4%	31.8 (15.6); 88.7%	< 0.001
Push-ups (repeats); HFZ (%)	10.9 (7.3); 66.6%	13.9 (8.2); 64.7%	8.20 (5.2); 68.2%	< 0.001
Sit and reach (cm); HFZ (%)	24.8 (6.7); 57.2%	22.9 (6.5); 77.6%	26.5 (6.5); 39.3%	0.363
Pacer 20 m (laps); HFZ (%)	39.6 (19.5); 78.5%	49.6 (21.5); 89.7%	30.8 (11.9); 68.7%	< 0.001
Sedentary time (min/day)	498.3 (94.0)	482.8 (96.9)	512.0 (89.3)	< 0.001
Prolonged SB (bouts/day)	1.0 (0.8)	1.1 (0.8)	1.0 (0.8)	0.205
Non-prolonged SB (bouts/day)	120.3 (16.6)	119.3 (16.2)	121.1 (16.8)	0.767
Breaks in SB (breaks/day)	87.5 (13.9)	86.9 (13.7)	87.9 (14.2)	0.372
LIPA (min/day)	302.7 (78.6)	308.9 (80.3)	297.3 (76.7)	0.265
MVPA (min/day)	37.3 (22.0)	45.9 (23.9)	29.7 (17.1)	< 0.001

*p-*value for the *T*-test in continuous variables, and the qui-square test to compare differences in proportions between boys and girls

*n* number of participants, *SD* standard deviation, *HFZ* healthy fitness zone, *SB* sedentary behavior, *BMI* body mass index, *LIPA* light intensity physical activity, *MVPA* moderate-to-vigorous physical activity, *PACER* progressive aerobic cardiovascular endurance run

The correlations between all the main independent covariates and multicollinearity tests are shown in Table 2. The VIF was <5.0 suggesting low possibility for multicollinearity between exposure variables. Similarly, the correlation coefficients between all the independent variables were <0.75 indicating low risk for collinearity.

**Table 2:** Correlations for the main covariates and multicollinearity

	Sedentary time	Prolonged SB	Non-prolonged SB	Breaks in SB	MVPA	VIF
Sedentary time (min/day)	1.000	0.017	**0.278**	−**0.143**	−**0.345**	1.494
Prolonged SB (bouts/day)	0.017	1.000	**0.069**	0.007	0.008	1.008
Non-prolonged SB (bouts/day)	**0.278**	**0.069**	1.000	**0.651**	**−0.165**	2.318
Breaks in SB (breaks/day)	**−0.143**	0.007	**0.651**	1.000	**−0.039**	2.180
MVPA (min/day)	**−0.345**	0.008	**−0.165**	**−0.039**	1.000	1.146

The Pearson correlation coefficients in bold mean significant correlations (*p ≤* 0.05)

*SB* sedentary behavior, *MVPA* moderate-to-vigorous physical activity, *VIF* variance inflation factor. The VIF for each independent variable was calculated to evaluate multicollinearity

The associations for total sedentary time, MVPA, breaks in sedentary time, non-prolonged and prolonged sedentary bouts with standardized fitness composite score (z-score) are shown in Table 3. Interactions between sex and the independent variables (MVPA, total sedentary time, breaks in sedentary time, prolonged and non-prolonged sedentary bouts) were tested. Because a significant interaction between sex and MVPA (*p =* 0.031) in the association with the dependent variable was found, all further analyses were stratified by sex.

**Table 3:** Association for sedentary time, MVPA, breaks in sedentary time, non-prolonged and prolonged sedentary bouts with standardized fitness composite score (z-score), by sex

	Fitness composite score					
	All		Boys		Girls	
	β (95% CI)^a^	β (95% CI)^b^	β (95% CI)^a^	β (95% CI)^c^	β (95% CI)^a^	β (95% CI)^c^
Sedentary time (min/day)	0.000 (−0.001; 0.002)	0.001 (−0.001; 0.002)	0.00 (0.000; 0.004)	0.001 (−0.002; 0.003)	−0.001 (−0.003; 0.000)	0.000 (−0.001; 0.002)
Prolonged SB (bouts/day)	0.080 (−0.064; 0.223)	0.080 (−0.063; 0.223)	0.167 (−0.065; 0.398)	0.132 (−0.096; 0.360)	0.001 (−0.177; 0.178)	0.081 (−0.094; 0.256)
Non-prolonged SB (bouts/day)	**0.010 (0.004; 0.017)**	**0.011 (0.002; 0.020)** ^**d**^	0.011 (0.000; 0.022)	0.003 (−0.013; 0.018)	**0.010 (0.002; 0.018)**	**0.014 (0.003; 0.024)** ^**d**^
Breaks in SB (breaks/day)	**0.009 (0.001; 0.017)**	**0.024 (0.013; 0.034)** ^**d**^	0.008 (−0.005; 0.021)	**0.026 (0.009; 0.042)** ^**d**^	**0.010 (0.001; 0.020)**	0.001 (−0.017; 0.019)
MVPA (min/day)	**0.008 (0.003; 0.013)**	**0.010 (0.005; 0.016)** ^**d**^	**0.008 (0.001; 0.015)**	**0.013 (0.005; 0.021)** ^**d**^	**0.011 (0.003; 0.019)**	**0.014 (0.006; 0.022)** ^**d**^

Data are unstandardized β coefficient and 95% confidence interval (CI)

*SB* sedentary behavior, *MVPA* moderate-to-vigorous physical activity

^**a**^Unadjusted model

^**b**^The models were adjusted for total sedentary time, sex, age, and season of data collection

^**c**^The models were adjusted for total sedentary time, age, and season of data collection

^**d**^Variables that remained in the model

β coefficients in bold mean significant associations (*p ≤* 0.05)

After adjusting for age, season of data collection, and total sedentary time, MVPA was positively associated with fitness composite scores in both girls and boys (*p <* 0.001). Breaks in sedentary time (*p <* 0.05) were positively associated with fitness composite scores after adjustment for age, season, total sedentary time, and MVPA in boys. Similarly, non-prolonged sedentary bouts (*p <* 0.05) were positively associated with fitness composite scores after adjustment for age, season, total sedentary time, and MVPA in girls.

Total sedentary time was not associated with fitness composite score in both unadjusted and adjusted models (*p >* 0.05) in both sexes. For each one-minute difference in MVPA, the fitness composite score was 0.014 higher, independently of total sedentary time and other covariates in girls. Similarly, each one-minute difference in MVPA was associated with 0.013 higher fitness composite score in boys. Additionally, each break in sedentary time was associated with 0.026 higher fitness composite score in boys.

The associations between total sedentary time, MVPA, breaks in sedentary time, non-prolonged and prolonged sedentary bouts with individual FITNESSGRAM® components are shown in Table 4. Total sedentary time was positively associated with curl-up test (*p <* 0.05) in girls. No associations were found for any of the covariates with the push-up test in girls (*p >* 0.05). MVPA and non-prolonged sedentary bouts were positively associated with flexibility (sit and reach) (*p <* 0.05). Similar associations were observed for the pacer test, with MVPA and non-prolonged sedentary bouts being positively associated, (*p <* 0.05). Finally, non-prolonged sedentary bouts but not MVPA was inversely associated with BMI in girls, (*p <* 0.05). All analyses were adjusted for age, season, and mutually adjusted for total sedentary time and/or MVPA.

**Table 4:** Association for sedentary time, MVPA, breaks in sedentary time, non-prolonged and prolonged sedentary bouts with FITNESSGRAM® tests, by sex

	Curl up	Push up	Sit and reach	Pacer	BMI
	β (95% CI)	β (95% CI)	β (95% CI)	β (95% CI)	β (95% CI)
Girls					
Sedentary time (min/day)	**0.015 (0.005; 0.025)** ^**a**^	0.000 (−0.004; 0.003)	−0.004 (−0.008; 0.000)	0.001 (−0.007; 0.009)	−0.001 (−0.003; 0.002)
Prolonged SB (bouts/day)	1.647 (−28.163; 31.458)	−2.925 (−13.273; 7.423)	−3.041; −15.559; 9.477)	10.453 (−12.206; 33.112)	−3.388 (−10.803; 4.028)
Non-prolonged SB (bouts/day)	−0.016 (−0.076; 0.044)	0.006 (−0.015; 0.026)	**0.049 (0.024; 0.075)** ^**a**^	**0.056 (0.010; 0.102)** ^**a**^	**−0.016 (−0.026; −0.006)** ^**a**^
Breaks in SB (breaks/day)	−0.037 (−0.119; 0.044)	−0.011 (−0.039; 0.017)	**−0.058; −0.093; −0.024)** ^**a**^	−0.047 (−0.109; 0.015)	−0.015 (−0.036; 0.005)
MVPA (min/day)	0.025 (−0.021; 0.071)	0.015 (−0.002; 0.031)	**0.025 (0.005; 0.044)** ^**a**^	**0.067 (0.032; 0.102)** ^**a**^	−0.005 (−0.016; 0.007)
Boys					
Sedentary time (min/day)	**0.013 (0.001; 0.026)** ^**a**^	0.005 (−0.001; 0.010)	−0.003 (−0.008; 0.002)	0.004 (−0.009; 0.017)	0.001 (−0.002; 0.004)
Prolonged SB (bouts/day)	−7.137 (−45.323; 31.050)	4.841 (−12.610; 22.292)	−12.069; −27.270; 3.846)	29.656 (−11.007; 70.319)	−2.422 (−11.041; 6.196)
Non-prolonged SB (bouts/day)	0.003 (−0.075; 0.081)	**−0.051 (−0.087; −0.015)** ^**a**^	0.024 (0.010; 3.132)	0.026 (−0.057; 0.110)	−0.013 (−0.031; 0.005)
Breaks in SB (breaks/day)	**0.075 (0.008; 0.141)** ^**a**^	**0.063 (0.019; 0.107)** ^**a**^	−0.018; −0.057; 0.020)	0.002 (−0.101; 0.105)	**−0.030 (−0.045; −0.015)** ^**a**^
MVPA (min/day)	−0.001 (−0.025; 0.169)	0.001 (−0.019; 0.018)	0.007 (−0.009; 0.023)	**0.093 (0.049; 0.136)** ^**a**^	**−0.017 (−0.026; −0.009)** ^**a**^

Data are unstandardized β coefficient and 95% confidence interval (CI). The models were mutually adjusted for total sedentary time and/or MVPA, age, and season of data collection

*SB* sedentary behavior, *MVPA* moderate-to-vigorous physical activity, *BMI* body mass index

^a^Variables that remained in the model

β coefficients in bold mean significant associations (*p ≤* 0.05)

Total sedentary time and breaks in sedentary time were positively associated with curl-up test in boys, (*p <* 0.05). MVPA was the only variable with positive associations for the pacer test in boys, (*p <* 0.05). Non-prolonged sedentary bouts were inversely associated and breaks in sedentary time positively associated with the push up test in boys, (*p <* 0.05). No associations were found for any of the covariates with flexibility (sit and reach) in boys, (*p >* 0.05). Finally, MVPA and breaks in sedentary time were inversely associated with BMI in boys, (*p <* 0.05). All analyses were adjusted for age, season of data collection, and mutually adjusted for total sedentary time and/or MVPA.

## Discussion

MVPA was positively and consistently associated with fitness in boys and girls, independent of sedentary time and patterns. Modest and inconsistent associations were found between sedentary behavior and fitness. Breaks in sedentary time was positively associated with fitness, independent of total sedentary time and MVPA in boys. In girls, non-prolonged sedentary bouts (<30 min) were positively associated with fitness, independent of total sedentary time and MVPA. These results suggest that encouraging young people to engage in more MVPA and reduce their sedentary time may have beneficial effects on their health related fitness.

Current guidelines for public health [38] suggest that youth should accumulate at least 60 min of MVPA each day, minimize sedentary time each day by limiting recreational screen time to no more than 2 h/day, and limiting sedentary (motorized) transport, extended sitting time and time spent indoors [38]. Additionally, an international study [39] concluded that youth spent 8.6 h per day sedentary, and simultaneously 54.2% of youth failed to meet sedentary behavior guidelines [39].

In accordance with the findings from a recent meta-analysis [14], no association was found for total sedentary time with fitness level in either boys or girls in the present study. Our findings suggest that specific patterns of sedentary time, e.g. the frequency of breaks in sedentary time, may be associated with higher fitness levels. This may then suggest that guidelines for sedentary behavior may include a recommendation to break-up sedentary time; effectively increasing overall physical activity. However, our findings need replication in future studies before implementation in public health recommendations for young people.

It can be argued that the benefits do not come from the breaking pattern itself, but rather the increase in LIPA, that derives from these breaks. However, previous studies found that total sedentary time and the amount of time spent in LIPA do not associate with fitness in youth [16, 40], suggesting that the positive association found for the number of breaks in sedentary time (while performing LIPA) in the present study are not a result of LIPA accumulation but rather reflecting the beneficial effect of an interrupting pattern. However, it is important to acknowledge that quality evidence from studies with robust designs and methods, such as experimental studies controlling for the total amount of sedentary time, matching PA between conditions by manipulating the breaking pattern across conditions, are needed to accurately test this hypothesis [17].

Less than 20% of children meet PA recommendations [41] and high intensity PA declines during youth [42]. Therefore, it is crucial to understand if the associations for MVPA and sedentary behavior with fitness levels are independent of each other. Contrarily to previous findings suggesting negative associations between sedentary time and fitness levels [11, 12], we did not observe an association between total sedentary time and the composite fitness score independent of MVPA suggesting total sedentary time is unrelated with fitness.

When analyzing the associations for each individual component of the fitness test, the observed associations for MVPA were less consistent. No significant associations were found between MVPA strength tests in both boys and girls, and with flexibility in boys, suggesting that MVPA may be associated with overall fitness in both sexes, through its influence on endurance fitness (PACER) [43].

Unexpected positive associations were found for total sedentary time with the curl-up test in both boys and girls. The curl-up test is an indicator of abdominal muscle resistant strength and there is evidence for a negative association between sedentary behavior and strength outcomes in youth [44]. There is no logical or physiological explanation for the unexpected associations found between sedentary time and strength. It is important to mention that in the present study the majority of the participants were within the healthy fitness zone (90.4%), presenting high values for this specific test. On the other hand, although significance was verified for this association, the magnitude of the association was low, and therefore any interpretation of the physiological significance of this observation should be cautious.

Positive associations were found for the non-prolonged sedentary bouts with flexibility, and endurance fitness (PACER), and with BMI (negative associations) in girls. In boys, breaks in sedentary time was positively associated with the overall composite fitness-score and all individual fitness tests, with the exceptions for the flexibility and PACER tests. The individual analysis for the main covariates with each of the specific fitness components suggest variable associations, showing that MVPA and sedentary patterns may play specific roles across the different fitness components. Moreover, muscular strength and endurance (curl-up and push-up tests) may be less affected by young people’s PA and sedentary patterns, compared with cardiorespiratory fitness (PACER test).

For each one-minute difference in MVPA the fitness composite score was 0.014 higher, independently of total sedentary time and other covariates, in girls. Similarly, each one-minute difference in MVPA was associated with 0.013 higher fitness composite score in boys. Moreover, each break in sedentary time was associated with 0.026 higher fitness composite score in boys, and for each non-prolonged sedentary bout, fitness composite score was 0.014 higher in girls. Regardless of the statistically significant associations found in our study, the magnitude of these associations was small and may not be clinically meaningful.

In the current educational systems, youth are indebted to be seated during the entire classes spending about 97% of the traditional classes sitting [45]. This represents at least 5-h of sitting at school with prolonged bouts in sedentary behavior. Therefore, regardless of the small clinical effects found in our results, scholar environment is a major opportunity to change sedentary patterns, because it entails a large opportunity to introduce LIPA breaks, which in the long-term may have significant impact on the energy balance preventing overweight and obesity as well as improving overall fitness levels of youth.

Recently, stand-up desks have been presented as a good external stimulus to reduce sitting time by shifting it for standing time in elementary school students [46]. However, the evidence for the benefits of prolonged standing in youth is still weak, and from an energetic point of view it has been shown to add very modest gain in young adults [47]. Based on the findings from the present study, it might be important to develop new strategies to reduce students’ prolonged sedentary time while at the school, additional to initiatives to primarily increase MVPA. Moreover, programs relying exclusively on reducing overall sedentary time may overlook an area that is of great importance to youth’s fitness. Along with messages related to accumulating at least 60 min · day^−1^ of MVPA, youth should be encouraged to break-up sedentary time during the day.

A potential sex dimorphism for the associations of breaks in sedentary time with fitness composite score may exist with boys benefiting the most through more breaks in sedentary time. Regardless of the positive associations found for the non-prolonged sedentary bouts with fitness composite score in girls, the non-significant associations for the breaks in sedentary time with fitness levels in girls seems to indicate that a higher rate of sedentary breaks may be more relevant to improve fitness composite score in boys. It is also important to highlight that in the present study it took a year to gather all the data. In fact, seasonal variation in PA and sedentary behavior seems to exist [48], and differences in temperature, rainfall can affect youth PA habits [48]. Thus, PA/sedentary patterns recorded in one specific week of that year may not represent a typical week, and so influence the associations for these covariates with fitness level. However, we further adjusted for the season of data collection, and the associations and their magnitude remained the same, with and without adjustment for this covariate.

### Strengths and limitations

This study is not without limitations. Regarding the prolonged and non-prolonged sedentary bouts, there are two major issues that could be responsible for some of the confusing findings and that need to be addressed. First, the low number of prolonged sedentary bouts that youth engaged and secondly the metrics, and how non-prolonged sedentary bouts were obtained. This variable includes any recorded period of continuous sedentary time with less than 30 min, which means that sedentary bouts of 5 or 25 min were both classified as non-prolonged sedentary bouts. When considering non-prolonged sedentary bouts, it was expected to find positive associations with fitness level, which was verified in girls. However, in boys, there was a negative association found for the non-prolonged bouts with the push-up test that can be possibly explained by this high interval (1 to 29 min of continuous sedentary time).

The cross-sectional nature of the data limits inference about the direction of causality for the associations found for the main PA and sedentary variables with fitness composite score. For example, we cannot rule out the possibility that more breaks in sedentary time or more non-prolonged sedentary bouts result from higher fitness levels in boys and girls respectively. Another major limitation was the absence of maturity assessment, a recognized confounder of great importance when investigating youth. However, the associations were controlled for age, which is a variable that accounts for some of the maturity’s variation.

An important strength of our study is that sedentary time was objectively measured by accelerometry in a large youth sample. Still accelerometers are not sensitive to detect all activities such as biking, standing, and upper-body movement, which may limit its applicability. Also, one alternative explanation for the counterintuitive findings related to the association between MVPA and fitness may be nothing to do with accelerometry per se, but instead one might hypothesize that concentrating on moderate and vigorous as a single indicator can blunt the association with fitness. Structured, systematic and usually vigorous exercise is usually needed to promote strength and speed, and MVPA may simply not measure this. Regardless, public health guidelines consider this dimension (MVPA) and also, in order to be able to compare with previous studies it was decided to consider MVPA.

## Conclusions

Moderate and vigorous PA is associated with fitness levels in youth, independent of total sedentary time and patterns. In addition, the present findings revealed inconsistent associations for sedentary behaviors with fitness levels. Therefore, this study suggests that promoting physical activity of at least moderate intensity is likely to positively influence on fitness in youth, and with less consistency, interrupting sedentary time may also represent an advantage.

## Abbreviations

BMI: Body mass index
CRF: Cardiorespiratory fitness
HFZ: Healthy fitness zone
LIPA: Low intensity physical activity
MVPA: Moderate and vigorous physical activity
PA: Physical activity
PACER: Progressive aerobic cardiovascular endurance run
VIF: Variance inflation factor
